# Deletion of the gene encoding the reductase component of 3-ketosteroid 9α-hydroxylase in *Rhodococcus equi* USA-18 disrupts sterol catabolism, leading to the accumulation of 3-oxo-23,24-bisnorchola-1,4-dien-22-oic acid and 1,4-androstadiene-3,17-dione

**DOI:** 10.1186/s12934-014-0130-3

**Published:** 2014-09-09

**Authors:** Chin-Hsing Yeh, Yung-Shun Kuo, Che-Ming Chang, Wen-Hsiung Liu, Meei-Ling Sheu, Menghsiao Meng

**Affiliations:** Graduate Institute of Biotechnology, National Chung Hsing University, 250 Kuo-Kuang Rd, Taichung, Taiwan 40227 ROC; Emeritus professor, Department of Biochemical Science and Technology, National Taiwan University, Taipei, Taiwan 10617 ROC; Graduate Institute of Biomedical Sciences, National Chung Hsing University, 250 Kuo-Kuang Rd, Taichung, Taiwan 40227 ROC

## Abstract

The gene encoding the putative reductase component (KshB) of 3-ketosteroid 9α-hydroxylase was cloned from *Rhodococcus equi* USA-18, a cholesterol oxidase-producing strain formerly named *Arthrobacter simplex* USA-18, by PCR according to consensus amino acid motifs of several bacterial KshB subunits. Deletion of the gene in *R. equi* USA-18 by a PCR-targeted gene disruption method resulted in a mutant strain that could accumulate up to 0.58 mg/ml 1,4-androstadiene-3,17-dione (ADD) in the culture medium when 0.2% cholesterol was used as the carbon source, indicating the involvement of the deleted enzyme in 9α-hydroxylation of steroids. In addition, this mutant also accumulated 3-oxo-23,24-bisnorchola-1,4-dien-22-oic acid (Δ^1,4^-BNC). Because both ADD and Δ^1,4^-BNC are important intermediates for the synthesis of steroid drugs, this mutant derived from *R. equi* USA-18 may deserve further investigation for its application potential.

## Background

**Figure 1:**
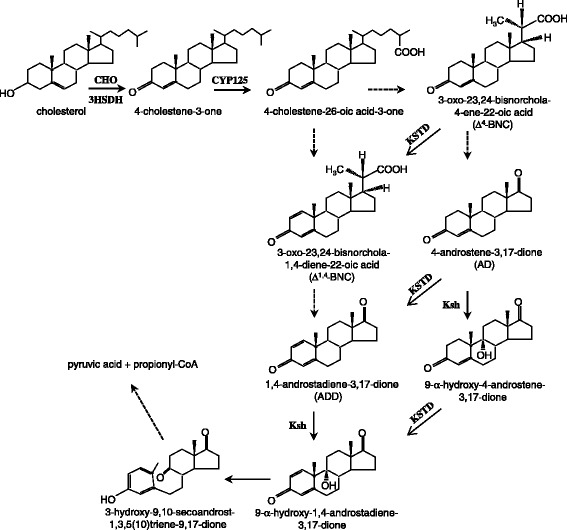
**The simplified illustration of the cholesterol catabolic pathway in** ***Rhodococcus*** **sp. CHO, 3HSDH, CYP125, KSTD and KSH denote cholesterol oxidase, 3-hydroxysteroid dehydrogenase, cytochrome P450 125, 3-ketosteroid Δ** ^**1**^ **dehydrogenase and 3-ketosteroid 9α-hydroxylase, respectively.** The arrow with dashed line denotes multiple enzymatic steps.

Steroid drugs, including androgens, anabolic steroids, estrogens and corticosteroids, have been widely used for a variety of health purposes. Currently, they are produced via chemical synthesis and/or biotransformation using steroid catabolic intermediates, such as 4-androstene-3,17-dione (AD) and 1,4-androstadiene-3,17-dione (ADD), as starting materials [1]. Bacterial strains belonging to *Mycobacterium*, *Rhodococcus*, *Nocardia* and *Arthrobacter* genera are known for their ability to degrade a range of naturally occurring steroids. The catabolic pathways for sterols have been proposed based on the results of a large body of biochemical and genomic studies [2–4]. A simplified illustration emphasizing biotransformation of AD and ADD is shown in Figure 1. The first step of the pathway is generally believed to be carried out by cholesterol oxidase, which catalyzes the oxidation of the 3β-hydroxyl moiety of sterols with the simultaneous isomerization of Δ^5^ to Δ^4^, resulting in the formation of 4-cholesten-3-one. However, recent studies on some *Mycobacterium* strains suggested that 3-hydroxysteroid dehydrogenase, rather than cholesterol oxidase, is responsible for the first step oxidation [5,6]. The degradation continues with side chain cleavage, taking place via a mechanism similar to β-oxidation of fatty acids, and polycyclic ring opening. These two processes were confirmed to be independent and the order of them varies among different bacterial strains [7]. In other words, the side chain degradation may occur at various points in the sterol degradation pathway. One possible route leads 4-cholesten-3-one to AD, which is then oxidized by 3-ketosteroid Δ^1^ dehydrogenase (KSTD) or 3-ketosteroid 9α-hydroxylase (KSH) to become ADD or 9α-hydroxy-4-androstene-3,17-dione (9OHAD), respectively. ADD and 9OHAD is then transformed into 9α-hydroxy-1,4-androstadiene-3,17-dione (9OHADD) by KSH and KSTD, respectively. 9OHADD is chemically unstable, undergoing spontaneous transformation into 3-hydroxy-9,10-secoandrost-1,3,5(10)-triene-9,17-dione (3HSA), a compound with an open B ring. Eventually, 3HSA is catabolized into pyruvate and propionyl-CoA.

Industrial processes use mutant microbial strains or add chemicals to inhibit the relevant enzymatic activities to accumulate AD, 9OHAD or ADD in the culture medium [8]. Modifying the sterol degradation pathway by genetic engineering may provide an alternative to allow the creation of potent strains for the production of those catabolic intermediates. Recent completions of the genomic sequences of bacterial strains such as *R. erythropolis* XP [9], *R. jostii* RHA1 [10] and *R. equi* [11] can provide valuable information when the rationally genetic approach is undertaken. Among the genes related to sterol catabolism, *KSTD* and *KSH* represent the most interesting targets for gene disruption because of their critical roles in the ring opening. Two KSTD isozymes were found in *R. erythropolis* SQ1 [12,13]. Loss of these two *KSTD* genes disabled *R. erythropolis* SQ1 from growing on steroid substrates; however, the mutant strain could efficiently convert AD into 9OHAD. Deletion of the *KSTD* gene in *M. neoaurum* NwIB-01 resulted in accumulation of AD from soybean phytosterols in the culture medium [14]. KSH is a two-component iron-sulfur-containing monooxygenase, consisting of the terminal oxygenase component (KshA) and the reductase component (KshB) [15–17]. Three KshA isozymes (KshA1 ~ KshA3) and one KshB have been found in *R. erythropolis* SQ1 [18]. The mutant SQ1 strain with either *KshA1* or *KshB* deletion was unable to grow in medium containing AD or ADD as the sole carbon and energy sources. Intriguingly, the mutant strain with null *KshB* was impaired in sterol degradation, suggesting that KshB of *R. erythropolis* SQ1 plays a role in not only ring opening but also in side chain degradation [17].

*A. simplex* B-7 was isolated as a cholesterol oxidase-producing strain from soil in Taiwan [19]. Cholesterol oxidase productivity of this *Arthrobacter* strain had been improved via UV-induced mutagenesis by 9 fold [20] and further increased via protoplast fusion by 20-60% [21]. A cholesterol oxidase gene, with accession number AY963570, was cloned from one of the improved strains, and its nucleotide sequence shows 99.6 and 98.9% identity to that of *R. equi* 103S and *Brevibacterium sterolicum*, respectively [22]. In order to learn more about the enzymes critical for cholesterol catabolism in strains derived from *A. simplex* B-7 and to explore the feasibility of producing AD or ADD by using genetically modified strains, we set out to clone *KshA* and *KshB* genes from *A. simplex* USA-18, a UV-induced mutant derived from *A. simplex* B-7 [20], in this study. Two hypothetical *KshA* and one putative *KshB* genes were cloned by PCR using degenerate primers, followed by inverse PCR and DNA walking. Subsequently, the effect of the putative *KshB* deletion on sterol catabolism was investigated. In addition, the 16S ribosomal RNA sequence and the metabolic profile of *A. simplex* USA-18 suggested that the strain should be renamed as *R. equi* USA-18.

## Materials and methods

### Chemicals

Cholesterol was purchased from Tokyo Kasei Kogyo Co., Ltd (Japan). Phytosterols, isolated from soybean, were purchased from a local food ingredient supplier (Ngya, Taiwan). AD and ADD standards were purchased from Sigma-Aldrich (USA).

### Bacterial strain

The gene deletion mutant, *R. equi* USA-18ΔB8, created in this study was deposited in the Bioresource Collection and Research Center (BCRC, Hsinchu, Taiwan) with stock number BCRC 910611.

### Culture condition

The bacterial strains used in this study were regularly grown in Luria-Bertani (LB) medium at 37°C. For the production of steroid metabolites, the cells were grown in glycerol minimal medium (1% (v/v) glycerol, 40 mg/L thiamine HCl, 4.65 g/L K_2_HPO_4_, 1.5 g/L NaH_2_PO_4_ · H_2_O, 3 g/L NH_4_Cl, 1 g/L MgSO_4_ · 7H_2_O, and 0.1% (v/v) trace element solution) supplemented with 2 g/L sterols (cholesterol or phytosterols) and 0.5% (v/v) detergent (Tween 20 or 80). Trace element solution contained 10 g ethylenediaminetetraacetic acid, 4.4 g ZnSO_4_ · 7H_2_O, 1.47 g CaCl_2_ · 2H_2_O, 1 g MnCl_2_ · 7H_2_O, 1 g FeSO_4_ · 7H_2_O, 0.22 g (NH_4_)_6_Mo_7_O_24_ · 4H_2_O, 0.315 g CuSO_4_ · 5H_2_O and 0.32 g CoCl_2_ · 6H_2_O in final 1 L water. To measure the cell density in the sterol-containing broth, an aliquot of the broth was added with glycerol to a final 50% concentration (v/v), thoroughly mixed, and subjected to 15000 rpm centrifugation. After removing the residual sterol that floated on the top, the cell pellet was suspended in a same volume of medium and the resulting optical density was measured by spectroscopy.

### Taxonomic determination

The nomenclature of the working strain was confirmed by the Biolog identification system (Biolog, USA)*.* Briefly, the bacterial colonies obtained after 36 h at 37°C on LB agar were transferred into the IF-A solution according to the manufacturer’s procedure. A 100-μl aliquot of the bacterial suspension was used to inoculate each of the culture wells of GEN_III_ MicroPlats. The metabolic profile after incubation at 30°C for 5 to 27 h was read by using Biolog’s Microbial Identification Systems software (OmniLog® Data Collection).

### Gene cloning

Sequences of the degenerate oligonucleotides, designed according to conserved motifs of the relevant enzymes, for initial PCR cloning are shown in Table 1. The 50 μL PCR solution contained 200 ng chromosomal DNA, specific degenerate primers (Table 1, each 100 μM), dNTPs (each 0.2 mM), 2.5 U *Pfu* polymerase, and 2% dimethyl sulfoxide. The PCR condition was 95°C for 3 min, 30 cycles of 94°C for 45 s, 55°C for 30 s, 72°C for 2 min, and a final step of 72°C for 10 min. The flanking sequences around the PCR-amplified regions in chromosome were obtained by inverse PCR [23] or DNA walking [24] using DNA Walking SpeedUp Premix kit (Seegene, Korea). The intact open reading frame of the cloned genes was finally amplified from chromosome DNA by PCR when sufficient information of the flanking sequences was available.

**Table 1:** **Primer pairs used in PCR in this study**

**Primer**	**Nucleotide sequence (5′ → 3′)**	**Product size (bp)**
#1: Asi9H1-S	CGNTAYGCNCGNGGNTGG	728
#2: Asi9H2-A	NACIGGRTARTGRCARTT	
#3: Asi9H3-S	TGYCCNTTYCAYGAYTGG	532
#2: Asi9H2-A	NACIGGRTARTGRCARTT	
#4: Asi9H7-S	GGNAGYGGIATHACNCC	530
#5: Asi9H5-A	CCYTCYCKRCAIGARTANGG	
#6: UpKshB-F	GGTAAGCTTACCGGTCGGCGAGCTCCTTGAACTCGTC	5212
#7: DnKshB-R	TCCGAATTCGGATCGACGCCCTCGGGCGGGACAC	
#8: Inv-F	TGTCGGTACCCACTCGGCACCCGGTCCCAGGAAGG	6669
#9: Inv-R	AGAATCTAGATTCTCGAGCGCGCGCACGAATCCTC	
#10: Kan-F	ATCTTCTAGAAGCTAGCTTCACGCTGCCGCA	1159
#11: Kan-R	ATTGAGGTACCCTCAGAAGAACTCGTCAAGAAGG	
#10: Kan-F	ATCTTCTAGAAGCTAGCTTCACGCTGCCGCA	2786
#12: Down-R	TCTCGTGAAGGAATTCGCGAACG	
#13: KshB-F	GAGCGGATCCGCTCCCGATCAGCGGGAGCCGGAA	2812
#12: Down-R	TCTCGTGAAGGAATTCGCGAACG	
#13: KshB-F	GAGCGGATCCGCTCCCGATCAGCGGGAGCCGGAA	1182
#14: KshB-R	CTGAAAGCTTTCAGAACTCGATCTTGAGGTGATCGGT	

### Gene deletion

An approximately 5.2-kb DNA fragment, encompassing the putative *KshB* in the middle, was obtained from chromosome of *A. simplex* USA-18 by PCR using primer 6 and 7 (Table 1), with *Hin*dIII and *Eco*RI sites engineered at the 5′ ends, respectively. The amplified fragment was inserted into *Hin*dIII-*Eco*RI-opened pUC18. The resulting plasmid, pUC-KshB5.2, was linearized by inverse PCR using primer 8 and 9 (Table 1), by which the open reading frame of the putative *KshB* was deleted. The kanamycin resistant gene plus its promoter (*Kan*), was amplified by PCR from pK18mobsacB (ATCC® 87097™) using primer 10 and 11 (Table 1). These two fragments were joined to form plasmid pUC-ΔΒ-Kan after both of them had been treated with *Kpn*I and *Xba*I. Aliquots (200 μL) of *A. simplex* USA-18 competent cells, suspended in 10% ice-cold glycerol, were mixed with 2 μg *Nde*I-treated pUC-ΔΒ-Kan in 2 mm gapped cuvettes. Electroporation was performed using the Gene Pulser apparatus (Bio-Rad, USA) under the condition of 12.5 kV/cm, 1000 Ω and 25 μF. After electroporation, the cells were incubated in 1 mL LB medium for 2 h at 37°C, 200 rpm, and subsequently plated on agar plates containing kanamycin (300 μg/mL). Colonies, appeared on the selection plates after about a week, were checked for homologous recombination by PCR using a sense primer matching *Kan* (primer 10) or *KshB* (primer 13) and an antisense primer (primer 12) recognizing a sequence downstream of the 5.2-kb *KshB*-containing fragment.

### Biotransformation product analysis

Culture broth was extracted with ethyl acetate at a ratio of 5:2 (v/v). Steroid metabolites in the extraction were regularly analyzed by thin layer chromatography (TLC) and high performance liquid chromatography (HPLC). TLC was performed using Kieselgel gel 60 F_254_ plates (Merck, Germany), developed in petroleum ether/ethyl acetate (6:4, v/v) solvent system, and visualized by spraying with 10% sulfuric acid and heating in a hot air oven at 120°C for 10 min. For HPLC, the metabolites were separated by Luna C18 column (250 x 4.6 mm, Phenomenex, CA) using 80% acetonitrile and 20% water as mobile phase at a flow rate of 1.0 mL/min, and detected at OD 254 nm. ADD concentration in the sample was calculated according to the standard curve of the known concentrations of ADD versus the respective peak areas in HPLC profile. Molecular weights of the metabolites and their fragmentation pattern were analyzed by ultra performance liquid chromatography-tandem mass spectrometry (UltiMate 3000 UHPLC, Thermo Scientific, USA) using ionization energy of 70 eV.

### Macrophage infection assay

The THP-1 human monocyte cell line was grown in RPMI 1640 medium supplemented with 10% fetal bovine serum (FBS), 0.45% glucose, 0.15% sodium pyruvate, 4 mM L-glutamine, and 1% PSA (penicillin-streptomycin-amphotericin B). One ml of 5 × 10^5^ cells/ml THP-1 cells was seeded onto each well of 6-well plates and the cells were treated with 300 nM phorbol 12-myristate 13-acetate for about 24 h to induce their differentiation to macrophages. The differentiated cells were washed with phosphate buffered saline (PBS) twice and re-suspended in antibiotic free RPMI 1640 supplemented with 2% FBS. The bacterial culture (10^8^ CFU/ml) was added into each of the wells at a multiplicity of infection of 10. After 1 h incubation, macrophages were washed twice with PBS to remove extracellular bacteria. The infected macrophages were then cultivated in RPMI 1640 supplemented with 10% FBS and 150 μg/ml ampicillin. At the indicated time points, the cells were washed with PBS twice and lysed by treating with 0.1% Tween 20 in PBS. The number of live bacteria released from lysed macrophages was determined by plate counting.

## Results

### Isolation of putative *KshA* and *KshB* genes from *A. simplex* USA-18

To clone *KshA* genes from *A. simplex* USA-18, the amino acid sequences of several bacterial Rieske [2Fe-2S] terminal oxygenases, including those isolated from *R. jostii* RHA1 (YP_704482), *M. smegmatis* (YP_890151), *Burkholderia cenocepacia* J2315 (YP_002234232), *Ralstonia eutropha* JMP134 (YP_295786) and *Comamonas testosteroni* KF-1 (WP_003057373), were aligned. Consensus motifs such as (R/T)(Y/F)(A/P)RGW and CP(F/Y)H(G/D)W were chosen for the design of sense degenerate primers (primer 1 and 3, respectively, Table 1), while the (N/I)(C/M)H(Y/V/T)P(I/V) motif was used for the design of an antisense degenerate primer (primer 2, Table 1). PCR using primer 1 and 2 gave rise to a 728-bp DNA (S1A2), while primer 3 and 2 produced a 532-bp DNA (S3A2). These two DNA fragments share 72.3% identity within the overlapped region, suggested that *A. simplex* USA-18 contains at least two potential *KshA* genes. The upstream and downstream regions of S1A2 were obtained by inverse PCR and DNA walking, and thus an S1A2-containing open reading frame (ORF) of 1155 nucleotides (accession number KJ598876) was identified. Searching databases using BLASTn algorithms revealed that the ORF exhibits 99.7% identity to a putative iron-sulfur binding oxidoreductase gene (*REQ_45190*) of *R. equi* 103S [11]. Similarly, the nucleotide sequences flanking S3A2 were determined and an ORF of 1161 nucleotides (accession number KJ598877) was identified. It shares 99.7% identity with another iron-sulfur binding oxidoreductase gene (*REQ_06790*) of *R. equi* 103S [11]. To clone *KshB* gene, the amino acid sequences of the reductase subunit of 3-ketosteroid 9α-hydroxylases from *M. smegmatis* (WP_003894254), *Pseudovibrio* sp. (WP_008550016), *R. erythropolis* (AAL96830), and *R. jostii* RHA1 (YP_705768) were aligned. Accordingly, the conserved GSGITP and PYSC(R/Q/K)(E/S)G motifs were chosen to design the sense and antisense degenerate primers, respectively (primer 4 and 5, Table 1). PCR using this pair of primer generated a 530-bp fragment (S4A5). A putative ORF of 1185 nucleotides (accession number KJ598878) was subsequently identified after the flanking regions of S4A5 were obtained by DNA walking. The 1185-bp ORF was found to have an identical nucleotide sequence to *REQ_36320* of *R. equi* 103S that presumably encoding the reductase component of 3-ketosteroid 9α-hydroxylase [11].

### Reclassification of *A. simplex* USA-18 as *R. equi* USA-18

The great resemblance of the genes cloned in this study and the cholesterol oxidase gene cloned previously [22] to those of *R. equi* 103S raised a suspicion of whether the taxonomic classification of *A. simplex* USA-18 had been properly determined. The gene encoding for 16S ribosomal RNA was amplified from *A. simplex* USA-18 by PCR using the universal primer 8 F and U1492R [25]. Blastn showed that the gene, with the accession number KJ598875, is highly similar, with identities over 99%, to the 16S ribosomal RNA genes isolated from a variety of *R. equi* strains. However, the identities between the gene and those from *Arthrobacter* strains are about 91-92%, suggesting that *A. simplex* USA-18 is phylogenetically closer to *R. equi* than to *A. simplex*.

To confirm the 16S ribosomal RNA sequence-based suggestion, the metabolic profile of *A. simplex* USA-18 was checked using the Biolog Identification System, in which the ability of the bacterium to metabolize 71 carbon sources and sensitivity to 23 chemicals were analyzed. The profile identifies the test strain USA18 as *Rhodococcus equi*, with similarity index between 0.774 and 0.789. Accordingly, *A. simplex* USA-18 was renamed *R. equi* USA-18 hereafter.

### Deletion of the *REQ_36320* ortholog in *R. equi* USA-18

**Figure 2:**
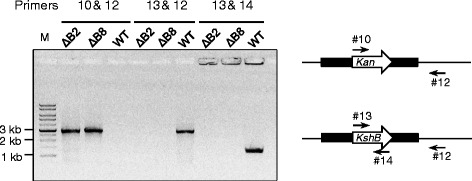
**Confirmation of the replacement of the putative chromosomal** ***KshB*** **with** ***Kan*** **by PCR.** Primer 10 specifically targets at *Kan*, while primer 13 and 14 at *KshB*. Primer 12 matches the downstream region of the putative *KshB*. The nucleotide sequences of the primers are shown in Table 1.

Searching protein homologs of the *REQ_45190* product within *R. equi* 103S using Blastp algorithm found another six potential Rieske [2Fe-2S] terminal oxygenases, which are products of *REQ_06790*, *REQ_08980*, *REQ_15470*, *REQ_40110*, *REQ_42740*, and *REQ_43730*. The amino acid sequence identities between the *REQ_45190* product and each of the homologs are 61.6, 57.3, 91.7, 63.4, 70.7, and 67.8%, respectively. None of their enzyme activities has been characterized. As to *REQ_36320*, no significant homolog was found. Considering the possible redundancy of *KshA* genes in *R. equi* USA-18, we chose the *REQ_36320* ortholog as the target for the activity disruption of 3-ketosteroid hydroxylase. Plasmid pUC-ΔΒ-Kan was constructed as described in materials and methods for the PCR-targeted gene disruption. The *Nde*I-linearized pUC-ΔB-Kan was introduced into *R. equi* USA-18 competent cells by electroporation. The colonies that survived on kanamycin-containing agar medium were further examined to assure the occurrence of a double crossover event in the flanking regions of the *REQ_36320* ortholog between chromosome and the introduced DNA by PCR. PCR amplification using primer 10 and 12 would generate a predetermined ~2.8-kb DNA product if the *REQ_36320* ortholog in chromosome had been replaced by *Kan* (Figure 2). PCR using primer 13 and 12 would otherwise generate another ~2.8-kb DNA fragment if the chromosome of *R. equi* USA-18 remained unchanged. Generation of the predefined PCR product in response to primer 10 and 12 but not to primer 13 and 12 suggests that the *REQ_36320* ortholog had been replaced with *Kan* in the chromosome of the two transformants (USA-18ΔB2 and USA-18ΔB8). PCR using *REQ_36320* specific primers (primer 13 and 14) confirmed the absence of the gene in these two transformants.

### Sterol catabolism in *REQ_36320* knockout mutant

**Figure 3:**
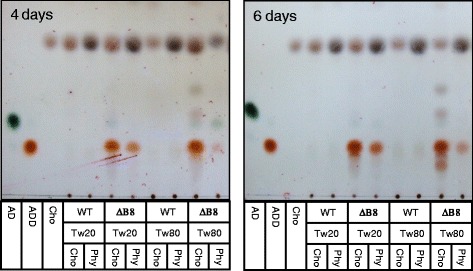
**Analysis of sterol metabolites by TLC.** *R. equi* USA-18 (WT) or *R. equi* USA-18ΔB8 (ΔB8) was cultivated in 0.2% cholesterol (Cho) or phytosterol (Phy) containing medium at 37°C, 200 rpm. Tween 20 (Tw20) or Tween 80 (Tw80) was included in the medium for sterol solubilization. The broth was harvested, at the indicated time, and extracted with ethyl acetate. The dissolved compounds were analyzed by TLC as described in Materials and methods. Cholesterol, AD, and ADD were used as standards.

*R. equi* USA-18 and *R. equi* USA-18ΔB8 were cultivated in medium that was supplemented with 0.2% (w/v) cholesterol or phytosterol and 0.2% Tween 20 or Tween 80. The broth, harvested at 4 and 6 days post-inoculation, was extracted with ethyl acetate and the compounds in the extract were analyzed by TLC (Figure 3). The parental strain USA-18 did not produce discernible metabolic intermediate of sterols. However, the knockout strain gave rise to a prominent spot having the same migration distance as ADD on TLC plate. Other minor substances in the extract of *R. equi* USA-18ΔB8 were noticed, particularly in the prolonged culture that contained Tween 80.

**Figure 4:**
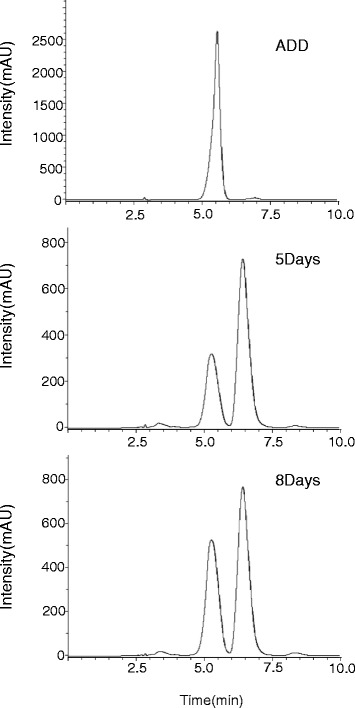
**Analysis of cholesterol metabolite by HPLC.** *R. equi* USA-18ΔB8 was cultivated in medium including 0.2% cholesterol and 0.2% Tween 20 at 37°C, 200 rpm. The broth, harvested at the indicated time, was extracted with ethyl acetate and the compounds within were analyzed by HPLC using a reverse-phase column as described in Materials and methods.

**Figure 5:**
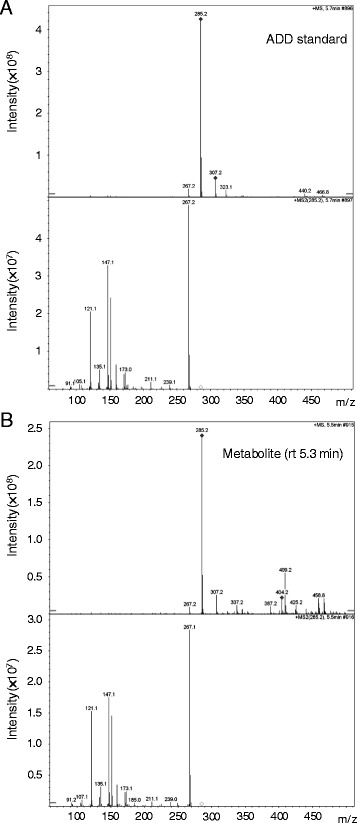
**Mass spectra of ADD standard and the cholesterol metabolite produced by** ***R. equi*** **USA-18ΔB8.** The strain was cultivated in medium containing 0.2% cholesterol and 0.2% Tween 20 at 37°C, 200 rpm, for 8 days. The broth was extracted with ethyl acetate and the extract was analyzed by HPLC and tandem mass spectrometry. **(A)** ADD standard, **(B)** the metabolite with retention time of 5.3 min in the HPLC profile (see Figure 4). The spectrum in the upper half of a panel indicates the molecular weights of the analyzed compounds, while that in the lower half shows the fragmentation pattern of the compound with 285.2 m/z.

To further identify the compounds in the spot that had the same migration rate as ADD on TLC, the culture broth was harvested at 5 and 8 days after inoculation, extracted with ethyl acetate, and analyzed with an analytical C18 reverse-phase HPLC column. Two peaks were found in the elution profile (Figure 4). The first peak has a retention time of ~5.3 min, approximately same as ADD standard, and its magnitude increased with the culture time. The second peak, with a retention time of ~6.5 min, remained relatively constant during the culture course. The chemical nature of the substance with the 5.3-min retention time was determined with UHPLC-MS/MS (Figure 5). This substance has a molecular weight of 284 Daltons, consistent with that of ADD. In addition, it generated a fragmentation pattern exactly identical to that of ADD. These data confirm that the substance, with the 5.3-min retention time, accumulated in the culture broth of *R. equi* USA-18ΔB8 is ADD. The molecular weight of the substance with the retention time of 6.5 min was also determined by mass spectrometry (data not shown). Its molecular weight was determined to be 342 Daltons. Presumably, this substance is Δ^1,4^-BNC, a precursor of ADD, according to its molecular weight and the fact that it was produced only when sterols were included in the culture medium.

**Figure 6:**
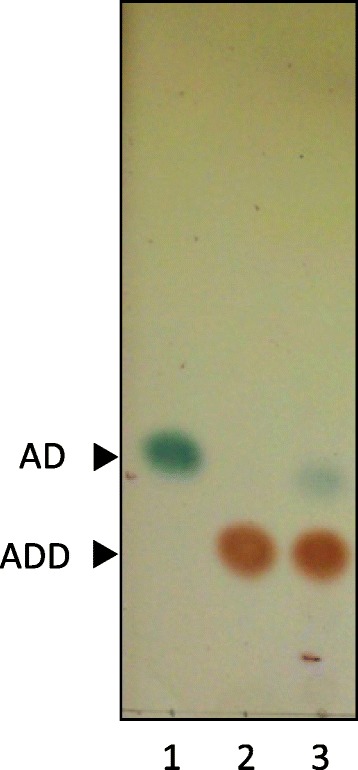
**Biotransformation of AD into ADD.** *R. equi* USA-18ΔB8 was cultivated in glycerol minimal medium supplemented with 0.5% (w/v) AD and 0.2% (v/v) Tween 20 at 37°C, 200 rpm, for 5 days. The broth was extracted with ethyl acetate and the dissolved compounds were analyzed by TLC. Lane 1 and 2 are AD and ADD standards, respectively. Lane 3 is the ethyl acetate extract.

Accumulation of Δ^1,4^-BNC in the broth suggests a catabolic pathway of sterol to ADD via Δ^1,4^-BNC (Figure 1). In other words, ADD not necessarily descended from AD after a dehydrogenation reaction. To know whether *R. equi* USA-18ΔB8 was capable of converting AD to ADD, the cells were cultivated in glycerol minimal medium supplemented with 0.5% (w/v) AD and 0.2% Tween 20 at 37°C for 5 days. TLC analysis indicated that most AD in the medium had been converted into ADD (Figure 6). Taken together, sterols are catabolized to ADD in *R. equi* USA-18 via at least two routes, namely from Δ^1,4^-BNC to ADD and from Δ^4^-BNC, AD to ADD.

### Time course of ADD production

**Figure 7:**
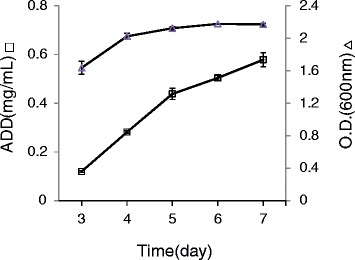
**Time course of** ***R. equi*** **USA-18ΔB8.** The bacterial strain was grown aerobically in glycerol minimal medium supplemented with 0.2% (w/v) cholesterol and 0.5% (v/v) Tween 20 at 28°C for 1 week. An aliquot of the broth was withdrawn every day. Cell density and ADD concentration of the samples were determined by the methods described in Materials and methods.

To assess the capability of *R. equi* USA-18ΔB8 for ADD production, the cells were cultivated in shaken flasks in glycerol minimal medium supplemented with 0.2% (w/v) cholesterol and 0.5% (v/v) Tween 20 at 28°C. An aliquot of the broth was withdrawn at daily intervals and the cell density and ADD within were determined. The cell density reached a plateau at about day 4, while the ADD concentration continuously increased to 0.58 mg/ml, equivalent to 40% molar yield, at day 7 (Figure 7).

### Restricted growth of *R. equi* USA-18 in macrophages

**Figure 8:**
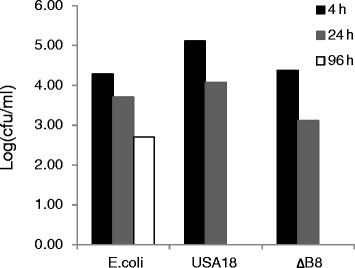
**Macrophage infection assay with** ***E. coli*** **Top10F'**, ***R. equi*** **USA-18, and** ***R. equi*** **USA-18ΔB8.** Macrophages derived from the human THP-1 cell line were infected with the indicated bacteria at a multiplicity of infection of 10 as described in Materials and methods. The number of intracellular bacteria within the macrophages was determined by plate counting at 4, 24, and 96 h post-infection.

Virulent strains of *R. equi* are recognized as facultative intracellular pathogens that cause severe pyogranulomatous bronchopneumonia in young foals. The pathogenic strains also cause an opportunistic infection in humans, particularly in HIV-infected and immunosuppressed patients [26]. Virulent plasmids, with the size up to 100 kb, are required for the virulent strains to survive within macrophages and for virulence in the susceptible hosts [27]. In addition, the sterol catabolic pathway is important for pathogenesis of *R. equi* [28]. *R. equi* is also a common soil-dwelling microorganism thriving on plant and animal sterols. *R. equi* USA-18 was originally isolated from soil [19]. No virulent plasmid was found in this strain. To determine whether *R. equi* USA-18 is of virulence, the growth of the strain within human macrophages was assayed as described in materials and methods. The cell number, cfu/ml, of *E. coli* Top10F' within macrophages continuously decreased during the incubation period, only 2.5% left at 96 h. As with the decrease of *E. coli* in macrophages, the viable counts of *R. equi* USA-18 and *R. equi* USA-18ΔB8 also decreased with time; however, they dropped to an undetectable level at 96 h. (Figure 8). Inability to persistently grow in macrophages suggests that *R. equi* USA-18ΔB8 has an application potential in industry.

## Discussion

This study cloned the *REQ_36320* ortholog from *R. equi* USA-18 and then deleted it to test whether its gene product participates in the 3-ketosteroid 9α-hydroxylase activity. An unmarked gene deletion approach using pK18mobsacB was taken on the first attempt. Unfortunately, *sacB*-carrying *R. equi* USA-18 was insensitive to sucrose up to 20% (w/v) (data not shown). The invalidity of using *sacB* as a counter-selection marker in *R. equi* was actually mentioned in literature [29]. A PCR-targeted gene replacement strategy, as described in materials and methods, was then launched to replace the *REQ_36320* ortholog with *Kan*. Dozens of colonies that were resistant to kanamycin were obtained after delivering 2 μg DNA construct into 1 × 10^9^ competent cells by electroporation. Of them, two colonies had the *REQ_36320* ortholog replaced correctly by *Kan*. This method thus proved to be an alternative for gene deletion in a *R. equi* type of strain.

*R. equi* USA-18ΔB8, the deletion strain that lacks the *REQ_36320* ortholog, survived on the medium that contained sterols as the sole carbon source and accumulated Δ^1,4^-BNC and ADD in the broth. However, this strain was unable to grow when ADD was supplied as the sole carbon source (data not shown); suggesting that the strain was devoid of 3-ketosteroid 9α-hydroxylase activity. It is logical to assume that the *REQ_36320* ortholog encodes the reductase component of 9α-hydroxylase based on the above observations and the similarity of its deduced amino acid sequence to the characterized reductase components isolated from *R. erythropolis* (78.9%) [17] and *R. rhodochrous* (78.3%) [16].

The reason why Δ^1,4^-BNC accumulated in the broth remains unclear. A mutant strain of *R. rhodochrous* DSM 43269, devoid of 3-ketosteroid 9α-hydroxylase activity due to inactivation of all the five *KshA* gene homologs, transforms cholesterol into ADD and Δ^1,4^-BNC in molar ratios of 3 and 73%, respectively [3]. Accordingly, accumulation of Δ^1,4^-BNC seems to be a common phenomenon in *Rhodococcus* species when their 3-ketosteroid 9α-hydroxylase activity is inactivated. This phenomenon implies that lack of the hydroxylase activity adversely affects the enzymatic steps leading Δ^1,4^-BNC to ADD. Since ADD could descend from either Δ^1,4^-BNC after side chain oxidation or AD after dehydrogenation at carbon 1and 2 (Figure 1), we propose that ADD in *R. equi* USA-18ΔB8 preferentially imposes a feedback inhibition on the enzymes involved in the side chain oxidation of Δ^1,4^-BNC, but not Δ^4^-BNC. After all, ADD and Δ^1,4^-BNC share a common polycyclic ring structure and this similarity may contribute to this presumed preference. Under this inhibition condition, the part of sterol that had been catabolized to Δ^1,4^-BNC would mostly stop at here, while the rest would be catabolized to ADD via the route involving Δ^4^-BNC and AD intermediates.

There are five putative KSTD isozymes in *R. equi* 103S according to Blastp analysis. It is logical to assume that each KSTD has its preferable 3-ketosteroids for dehydrogenation. In other words, transformation of Δ^4^-BNC, AD, and 9OHAD to Δ^1,4^-BNC, ADD, and 9OHADD (Figure 1), respectively, may be catalyzed by specific KSTD isozymes. It will be interesting to find out the number of KSTD isozymes in *R.equi* USA-18 and elucidate their specific roles in the sterol catabolic pathway.

*R. equi* USA-18ΔB8 was capable of converting sterol to ADD and Δ^1,4^-BNC. The molar yield of ADD was about 40% when 0.2% cholesterol was included in the culture medium in an uncontrolled small-scale cultivation system. Fermentation technology shall be employed to further evaluate the potential of the strain in the transformation of sterols to ADD or/and Δ^1,4^-BNC.
